# Rethinking the politics and implementation of health in all policies

**DOI:** 10.1186/2045-4015-2-17

**Published:** 2013-04-22

**Authors:** Matthias Wismar, David McQueen, Vivian Lin, Catherine M Jones, Maggie Davies

**Affiliations:** 1 European Observatory on Health Systems and Policies, Rue de l’autonomie 4, Brussels 1070, Belgium; 2 Atlanta, GA, USA; 3 La Trobe University, Melbourne, Australia; 4 Institut de Recherche en Santé Publique, Université de Montréal, Montreal, Canada; 5 Health Action Partnership International, London, UK

## Abstract

In Europe, successful health policies have contributed to a continued decline in mortality. However, not all parts of Europe have benefited equally and the sustainability of achievements cannot be taken for granted since health policies vary widely even among neighbouring countries. Furthermore, there are a number of remaining public health challenges such as food and alcohol polices. We argue that if we are to make further progress we need to rethink the politics and implementation of Health in All Policies. Commenting on an article analyzing the roll out and early implementation of Israel’s National Programme to Promote Active, Healthy Lifestyles provides an opportunity to thrash out four issues. First, intersectoral structures are key transmission belts for Health in All Policies between ministries and sectors and we need to exploit their specific uses and understand their limitations. Second, our analytical perspective should focus on what it takes to introduce policy change instead of assuming an idealized policy cycle. This includes a reconsideration of interventions which may not be very effective but help to raise the standing of health on the political agenda, thus providing a stronger basis for policy change. Third, we need to better understand variations in context between and within countries, e.g. why do some countries adopt Health in All policies but others don’t, and why is it that in the same country compliance with some health policies is better than with others. Finally, we will need to better understand how a diverse set of actors from other sectors can internalize health as an intrinsic value.

## 

In parts of the WHO European Region, the combined effects of economic growth, improved health care, and successful health policies (e.g., tobacco control, road traffic safety) have contributed to a continuous decline in mortality. These success stories, however, are contrasted by less favourable mortality trends in Eastern Europe and challenging issues in health policy, such as alcohol and food. Further progress cannot be taken for granted since neighbouring countries are developing in different directions [1].

If we are to tackle such challenging health policy issues, we need to get the politics and implementation of health policy right. We need to better understand what it takes to raise the standing of health issues on the political agenda, induce policy change and ensure sustainable implementation. This is why the article by Kranzler and colleagues [2] is very timely and of great importance. They analyze the recent roll out and early implementation of Israel’s National Programme to Promote Active, Healthy Lifestyles.

The National Programme’s main aim is to tackle obesity, a leading cause of ill-health and preventable death. It comes with ambitious, quantified goals. The key strategies to achieve these goals are increasing knowledge, fostering health-promoting environments and incentivizing organizations and municipalities to engage in health promotion. Attention is given to intersectoral structures for collaborating with ministries and stakeholders. The programme also includes a comprehensive legislative agenda.

The article raises a number of analytical and political issues worth commenting upon. First, intersectoral structures are key transmission belts when preparing, adopting and implementing Health in All Policies (HiAP). The authors have reported on some positive experience in Israel with the interdepartmental committee employed for the programme. However, beyond coordination, support seems to be lukewarm. Some authors have derided interdepartmental committees as an organizational mechanism and argued that they sometimes facilitate sabotage rather than progress [3]. If the issue the committees deal with is deemed unimportant or controversial, they will not work. We have recently reported on the failure of the English cabinet sub-committee on public health, which was abolished after only two years in existence [4]. We would argue that interdepartmental committees can only resolve administrative bureaucratic issues, and if they do not work then it is probably an expression of a lack of political support.

Second, to analyze the politics surrounding the adoption and implementation of the Israeli programme, the authors have adopted a non-linear model of policy making. According to this model, policy change does not progress in an orderly fashion from problem analysis, to decision making, to implementation and evaluation. On the contrary, problems, solutions and the politics are mostly disconnected. Policy entrepreneurs may provide us with solutions, before the problem is clear. Scientists identify problems but not solutions. Or we have problems and solutions acknowledged but they do not enter politics. This has consequences for the assessment of effective political strategies and tactics that have the potential to lift issues on the political agenda and prepare policy change.

For example, the literature does not provide much evidence on the effectiveness of private-public partnerships and self-regulation with regards to industry [5]. Discussing public health related food issues such as package size, labeling, and composition of ingredients with the industry can be frustrating and might only lead to small, temporary changes [6]. For example, the alcohol industry has promoted corporate social responsibility, a policy intervention that has been proven to be ineffective as the incentives favour irresponsibility rather than responsibility [7]. However, if an intervention that does not lead to an effective solution itself helps to lift the issue on the political agenda, thus helping to open a window of opportunity for much more effective regulation, then we should rethink the role of these interventions.

The complexity of policy making also reminds us that achievements can be reversed at any time. A host of counter-strategies have been employed by vested interests to avoid or subvert HiAP [8]. These include: casting doubt on scientific evidence and misleading the public by denying negative health effects; permeating and, at times, infiltrating other sectors or decision-making levels; abusing a participatory role as an actor in the policy arena; using litigation at national and international levels to challenge policy decisions; and creating alliances with other business sectors. In the case of the tobacco industry, for example, such practices include forging alliances with the hospitality, gambling, retail and advertising industries. Harmful products may be moved to countries with the least resistance, thereby compensating industry losses in those countries that implement Health in All policies. Industry strategies are global and we will need to have both the analytical and political strategies to confront the industries’ counterstrategies. International and supranational institutions could play a leading role in this.

A third point is that we need to think about the context and conditions for implementation and enforcement of different policies. We need to understand why in some countries Health in All Policies are enforced, while in other countries even existing legislation can be ignored. There are also variations within countries between different health issues and products. For example, smoking bans in public places are often fully respected while alcohol control legislation is ignored and violated without much consequence [9].

Fourth, the authors consider this new initiative to be a paradigm shift for Israel’s Ministry of Health. The Programme brings health promotion, a formerly marginal issue to the center of Israel’s health agenda. This paradigm shift coincides with the launch of the new WHO European Health Policy Health 2020 [10]. The policy emphasizes building on a whole-of-government and a whole-of-society approach with implications for developing new roles for the Ministry of Health but also for actors beyond government including providers, stakeholders and citizens. The issue of intersectoral governance is high on the agenda of this new policy. We suggest that this has two implications. One is that more analysis of politics and governance is needed to build strategies to strengthen accountability for health across government departments and society. The second is that we need to better understand how diverse actors such as government officials, private industry and citizens may internalize health as an important objective, as we all have internalized the importance of evidence, efficiency, integrity, anti-discrimination and many other values.

Kranzler and colleagues have provided an important analysis with many insights that will inform, and hopefully encourage, other countries and policy makers. We hope that this article is only a first installment that will be followed by analyses of the subsequent implementation efforts. And there is a lot to learn, since the next challenges are just around the corner: What needs to be done to keep up the momentum in a process, stretching inevitably over a long period? What can be done to ensure support in the population? What is to be done if the looming austerity budget threatens to reduce National Programme funding? How is it possible to avoid a situation in which the comprehensive legislative agenda gets stuck in petty political conflicts? How can the leaders of the initiative ensure that the political capital spent by the Minister and other leading officials will create enough return on investment to strengthen their position and with it their political clout in the cabinet and in interdepartmental negotiations? It would be more than welcome if further developments and analysis of the National Programme could provide some answers to these pressing questions, helping us rethink the politics and implementation of HiAP.

### Authors’ information

Matthias Wismar is the Senior Health Policy Analyst of the European Observatory on Health Systems and Policies, Brussels, Belgium.

David V McQueen is a Consultant in Global Health Promotion. He is also Immediate Past President of the International Union for Health Promotion and Education (IUHPE) and, until retirement, was Associate Director for Global Health Promotion, Centers for Disease Control and Prevention, Atlanta, Georgia, USA.

Vivian Lin is Professor of Public Health at the Faculty of Health Sciences, La Trobe University, Melbourne, Australia. She is vice-president for scientific affairs for the International Union for Health Promotion and Education (IUHPE) and is senior editor for health policy for Social Science and Medicine.

Catherine M Jones is a PhD student in Public Health, University of Montreal. Prior to this, she was Programme Director at the International Union for Health Promotion and Education (IUHPE), Paris, France.

Maggie Davies is Executive Director of the Health Action Partnership International (HAPI), London, United Kingdom. She was most recently the Principal Advisor on International Health Inequalities for the Department of Health for England. Maggie has also worked at the national level as Associate Director of Development for the National Institute of Health and Clinical Excellence.
